# Prediction of Self-Association and Solution Behavior of Monoclonal Antibodies Using the QCM-D Metric of Loosely Interacting Layer

**DOI:** 10.1021/acs.molpharmaceut.4c00656

**Published:** 2024-11-29

**Authors:** Yusra Rahman, Siddhanth Hejmady, Reza Nejadnik

**Affiliations:** Department of Pharmaceutical Sciences & Experimental Therapeutics, College of Pharmacy, University of Iowa, Iowa City, Iowa 52242, United States

**Keywords:** developability, colloidal stability, self-association, monoclonal antibodies, protein solution behavior, QCM-D, loosely interacting layer

## Abstract

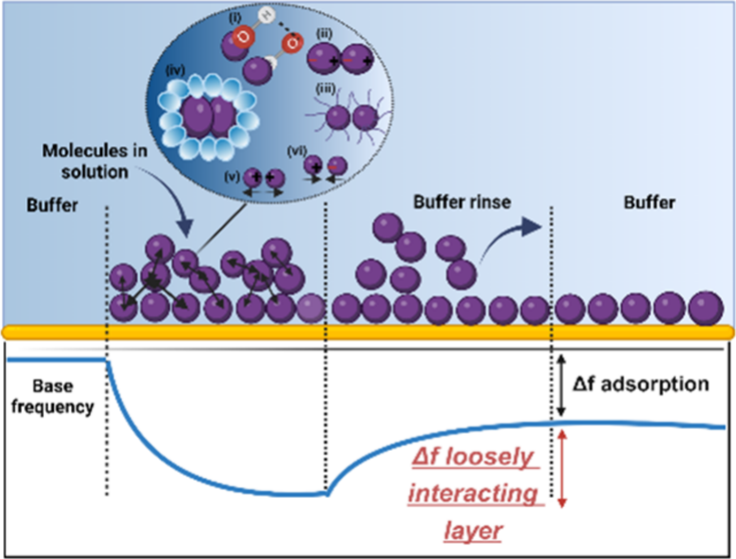

Despite the increasing availability and success of monoclonal antibodies (mAb), early identification of candidate molecules with desirable developability attributes remains challenging due to self-association and poor solution behavior. Measuring these phenomena experimentally using the available methods is complicated in mAbs development. Quartz crystal microbalance with dissipation monitoring (QCM-D) detects a loosely interacting layer on top of the irreversibly adsorbed layer of molecules, providing information about the mAbs interaction. This work aimed to explore whether the characteristics of this layer can be used as a reliable self-association metric. QCM-D experiments showed a large frequency shift (Δ*f*) associated with loosely interacting layers for omalizumab but a small or absent layer for tocilizumab. Accordingly, the viscosity of omalizumab increased exponentially at high concentrations compared to tocilizumab. Testing eight mAbs with different self-association behaviors revealed a strong rank order correlation between the mostly used metric of self-association, i.e., diffusion interaction parameter (kD-DLS), and Δ*f*, indicating Δ*f’*s potential for predicting mAb solution behavior. The study also highlighted the robustness of the metric to impurities and temperature variations compared to the sensitive kD-DLS. Overall, we demonstrate that the loosely interacting layer provides valuable information about mAb self-association, predicting the colloidal stability and solution behavior in therapeutic development.

## 1 Introduction

The use of monoclonal antibodies (mAbs) in treating a wide array of human diseases has increased immensely owing to their high affinities, exquisite specificity, superior safety, and extended half-lives. However, despite more than 100 approved products,^1^ the development of therapeutic mAbs remains an arduous task with a high failure rate.^2^ Therefore, the focus of the work in discovery and development has expanded to areas beyond biological function and is turned particularly to identifying protein candidates with optimal manufacturability, stability, and delivery profiles, a suite of characteristics that are known as developability attributes.^3^ A recent key study validated a selection of 12 developability metrics versus 137 antibodies that are already approved or are in advanced clinical stages and found that the number of “red flags” based on developability assessments was lower for the molecules furthest along in the development process and lowest for the approved ones.^4^ In our opinion, this observation is extremely interesting and indicates that mAbs that made it into the market or advanced stages of development did not reach there only because of their outstanding biological function (although that has to be a part of it) but because they were developable. Thus, there is a critical need to comprehensively understand the developability profile of protein molecules to optimize the selection of candidates with the best chance of success in drug development. One of the most important developability attributes is solubility and colloidal stability of the protein, in which protein self-association plays a key role. Self-association in this context refers to the net-attractive sum of all of the weak, noncovalent forces that can potentially bring these molecules together while in solution. High self-association and poor solution behavior have led to complications in the development of mAbs^4^ and high viscosity, opalescence, phase separation, and aggregation have been reported to be among the immediate problems, while overall long-term stability is hard to achieve when proteins self-associate.^5−12^ It is therefore imperative to have methods that identify variants of a molecule with favorable solution behavior during developability studies before advancing a candidate to clinical development.^13^

Methods that measure self-association are, however, limited, and those that are used are mostly based on complex, indirect measurements that cannot be performed under relevant conditions. Affinity capture self-interaction nanoparticle spectroscopy (AC-SINS),^14−16^ or its derivatives such as CS-SINS,^17,18^ which work by capturing the mAb on the surface of antihuman IgG-coated gold nanoparticles, allow for measuring self-association of antibodies by detecting nanoparticles aggregation. These methods, especially the latter, are highly sensitive and can be used with ultralow concentration samples, requiring only a small amount of protein. This makes them particularly useful during the discovery stages of protein development. However, these approaches are limited in that they require the development and use of specialized nanoparticles and that they may not be easily compatible with formulations of different buffers, pHs, and excipient compositions. Similarly, standup monolayer adsorption chromatography (SMAC) has been used to relate the retention time of protein in the column with its self-association and aggregation propensity.^19^ However, the application of this method depends on the availability and useful life of the specialized SMAC column, which will in turn depend on the nature of the mAbs employed in the study as impure or sticky proteins accelerate the decay of the column. In another approach, mass-dependent separation of assemblies in the centrifugal field to study ultraweak binding, using a sedimentation velocity technique, has been applied to identify self-associating proteins.^20^ These approaches are complex and require the employment of instrumentation, coatings, or chromatography columns that need additional development, making the methods not readily available for widespread application. Diffusion interaction parameter (kD-DLS) and osmotic second virial coefficient (B2) have been calculated using a series of dynamic and static light scattering (DLS and SLS) measurements and used to quantify self-association.^21,22^ Notably, Kingsbury et al. studied the kD-DLS for a set of 59 mAbs and showed that kD-DLS can predict the solution behavior of antibodies as represented by viscosity and opalescence at high concentration (150 mg mL^–1^, which is relevant for mAbs that are administered subcutaneously).^23^ Our data, however, suggest that the kD-DLS measurements are associated with large errors and that the metrics obtained by these methods are highly susceptible to temperature changes and the presence of impurities, including protein aggregates. In addition, for a typical kD-DLS measurement, mAbs are dissolved in a single buffer and a fixed pH in the absence of all of the relevant excipients due to the limitations of these methods, suggesting that these methods cannot measure protein–protein interactions under relevant conditions (the actual formulations that are used to store protein therapeutics). Thus, multiple reports highlight the discrepancies between the outcome of these assessments and have raised questions as to the validity of using these methods for understanding protein self-associations and their relation to viscosity and colloidal stability.^5,24−29^

**Figure 1:**
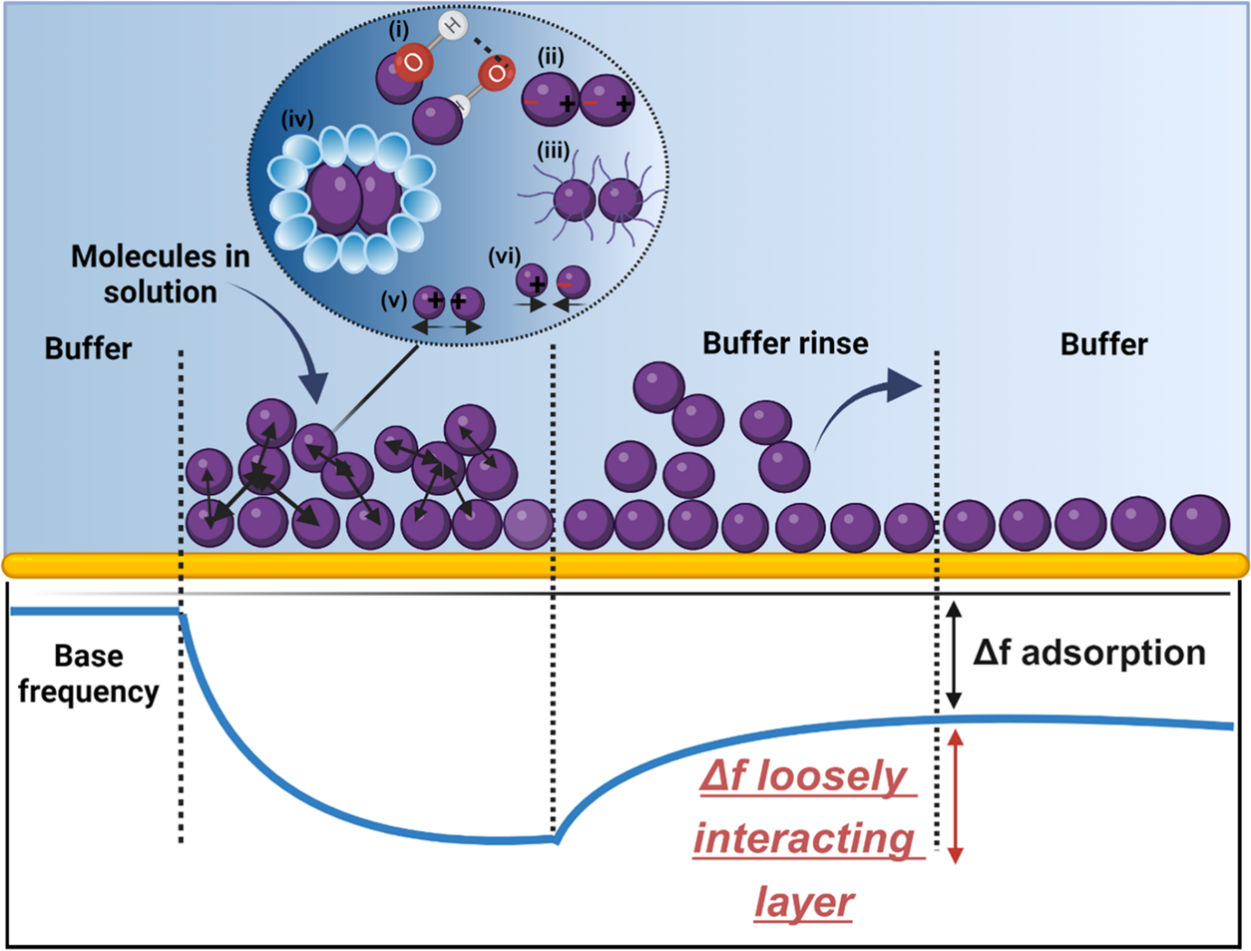
Schematic representation of the hypothesis of our work depicting the adsorption of molecules on the hydrophilic gold surface as detected by QCM-D. The participation of several intermolecular forces in the loosely interacting layer formed on top of the adsorbed layer. The forces that participate in forming the layer are as follows: (i) hydrogen bond, (ii) van der Waals’ interaction, (iii) steric repulsive forces, (iv) hydrophobic interactions, (v) repulsive charge–charge interaction, (vi) attractive charge–charge interactions. Herein, our novel approach focuses on the assessment of frequency shifts associated with the unexplored loosely interacting layer that would be influenced by the self-association of proteins in this layer and their interaction with the irreversibly bound protein layer adsorbed on the surface of the sensor as per our hypothesis.

We hypothesized that analysis of the crowded protein layer at the proximity of a solid surface using a sensitive adsorption analysis would provide a valuable metric for assessing the self-association of proteins. Quartz crystal microbalance with dissipation monitoring (QCM-D) is an established technique that provides real-time information on the adsorption of molecules to solid surfaces by monitoring the change in the resonance frequency of an oscillating piezoelectric sensor.^30^ The amplitude of the oscillations is influenced by dissipative energy losses caused by the viscoelastic properties of the adsorbed molecules, which can be quantified by measuring the changes in the frequency bandwidth or the decay of the induced oscillations. Over the past decade, the application of QCM-D has been expanded to innovative approaches in studying various biomolecular/nanomaterial bindings^31^ and adsorption phenomena.^32−34^ A typical adsorption experiment using QCM-D consists of three main steps: (a) a baseline frequency and dissipation signal is obtained by flowing the buffer/vehicle and (b) solution/suspension of molecules of interest are introduced and the adsorption process begins. Added mass of the sensor (due to adsorption) produces a negative frequency shift Δ*f*, while any potential softness of the adsorbed layer produces a positive dissipation change Δ*D*, until the surface is saturated, and (c) a rinse step with buffer/vehicle is performed to remove unbound molecules (Figure 1). The adsorbed layer often refers to the layer of molecules that is irreversibly bound and remains on the surface after a rinse. QCM-D can, however, detect some loosely interacting molecules on top of the adsorbed layer, which are rinsed away by the flow of a buffer. Several studies have observed this layer and reported considerable frequency and dissipation shifts associated with this layer and papers that mention the loosely interacting layer or similar terms have been enlisted (Table 1). Despite multiple reports, this loosely interacting layer has not been linked to the physical phenomena of self-association, and the potentially important information that it carries has never been utilized. The analysis of the listed papers reveals that only four papers discussed this layer beyond mere observation. For instance, Vogler states that a proper adsorption profile would require a collective assessment of both strongly and loosely bound layers.^35^ Also, Oom reported a secondary observation that between two studied proteins the one that had a higher propensity to aggregate showed a thicker loosely interacting protein layer in QCM-D studies. In this study, the trend has been reported for higher concentration samples (≥50 mg mL^–1^), that in the presence of self-association would have a higher viscosity.^36^ In line with this observation, other two studies by Patel et al. and Hartl et al. showed that the QCM-D signal can be used to estimate the mechanical properties of the viscous, high-concentration samples in contact with the sensor.^37,38^ Herein, our novel work takes a unique approach involving the assessment of frequency shifts and dissipation changes associated with the unexplored loosely interacting layer for low-concentration samples, which, according to our hypothesis, would be governed by the self-association of proteins in this layer and their interaction with the irreversibly bound protein layer adsorbed on the sensor surface. We demonstrate that the layer of loosely interacting molecules bears unique and practical insights into the self-association of the molecules for the first time, going beyond the understanding of protein adsorption and aggregation. By highlighting this previously overlooked aspect, our work adds a new dimension and a fresh perspective via the use of QCM-D as a powerful method to predict solution behavior in various applications in colloidal science and biotechnology.

**Table 1:** List of Papers Mentioning the “Loosely Interacting Layer”, “Loosely Bound Layer”, “Reversibly Adsorbed Layer”, “Adsorption Reversibility”, “Reversible Adsorption”, or Similar Terms That Have Been Used to Refer to the Loosely Interacting Layer as Described in the Literature with Information about the Discussion of This Layer in the Paper

serial no.	year of publication	title of paper	description of loosely interacting layer	DOI
1	2005	Adsorption of fibrinogen on tantalum oxide, titanium oxide, and gold studied by the QCM-D technique	The loosely bound layer formed in the case of high concentration with more water content has larger frequency shifts and a lower Δ*D*/Δ*f* value than the compact layer with low concentration.	10.1016/j.colsurfb.2005.04.007
2	2007	Probing protein adsorption onto mercaptoundecanoic acid stabilized gold nanoparticles and surfaces by quartz crystal microbalance and ζ-potential measurements	The thickness of the reversibly bound layer, for the adsorption of different proteins on self-assembled monolayers of mercaptoundecanoic acid (MUA)-coated gold surfaces and particles, increased with increasing protein concentration.	10.1021/la063725a
3	2007	Adsorption and viscoelastic properties of fractionated mucin (BSM) and bovine serum albumin (BSA) studied with quartz crystal microbalance (QCM-D)	The loosely interacting layer regarding the adsorption of bovine submaxillary gland mucin (BSM) on a hydrophobic surface was compared to the adsorption of BSA and the mixture of the two proteins.	10.1016/j.jcis.2007.07.029
4	2008	Thermal stability, mechanical properties, and water content of bacterial protein layers recrystallized on polyelectrolyte multilayers	The loosely bound layers of a bacterial protein recrystallized on charged monolayers incorporating 68% water.	10.1039/B719408K
5	2008	QCM-D sensitivity to protein adsorption reversibility	The loosely interacting layer is formed in the presence of high protein and low ammonium sulfate concentrations and larger dissipation is accompanied by forming a more reversibly adsorbed layer, probably conserving the native structure.	10.1002/bit.21977
6	2008	Viscoelastic characterization of high-concentration antibody formulations using quartz crystal microbalance with dissipation monitoring	A shift in frequency and dissipation was reversible but more attributed to changes in the viscosity of the solution in high-concentration protein formulations.	10.1002/jps.21610
7	2010	Adsorption from saliva to silica and hydroxyapatite surfaces and elution of salivary films by SDSand delmopinol	The loosely interacting layer, with respect to the adsorption of saliva to different surfaces, is considered to occur in a biphasic manner with the formation of an inner rigid layer followed by the formation of a loosely bound second layer with larger dissipation.	10.1080/08927014.2010.506609
8	2010	Adsorption of human serum albumin (HSA) on modified PET films monitored by QCM-D, XPS, and AFM	The steeper slope of the curve of the hydrolyzed PET surface (loosely interacting layer) leads HSA to be loosely bound to it compared to the normal PET surface.	10.1016/j.colsurfa.2010.03.003
9	2011	Surface interactions of monoclonal antibodies characterized by quartz crystal microbalance with dissipation: Impact of hydrophobicity and protein self-interactions	The formation of a loosely bound layer occurred in the case of mAb with higher concentration and the mAb with higher aggregation propensity showed a more pronounced loosely interacting layer in a study of the adsorption of two different behaving mAbs on polystyrene and Teflon surfaces.	10.1002/jps.22771
10	2011	Probing fundamental film parameters of immobilized enzymes—toward enhanced biosensor performance. Part I—QCM-D mass and rheological measurements	Using SDS removed the loosely bound protein adsorbed onto the surface to quantify the mass of covalently bound proteins to the electrode surface.	10.1016/j.enzmictec.2011.05.011
11	2012	Protein adsorption in three dimensions	The loosely interacting layer of protein is referred in context to protein adsorption to the surfaces, which requires collective assessment of both loosely and irreversibly bound layers.	10.1016/j.biomaterials.2011.10.059
12	2014	QCM-D study of layer-by-layer assembly of polyelectrolyte blend films and their drug loading-release behavior	The loosely interacting layer of protein on the surfaces is related to the larger dissipation shifts while studying the layer-by-layer formation of polyelectrolyte blend films and their drug-loading release behavior.	10.1016/J.COLSURFA.2013.10.033
13	2015	Adsorption and viscoelastic analysis of polyelectrolyte–surfactant complexes on charged hydrophilic surfaces	The loosely interacting layer is described in context to forming a diffused overlayer in the case of premixed solutions while comparing the adsorption between cationic polysaccharide and anionic solution of SDS on an amphoteric alumina surface.	10.1021/la5043052
14	2015	Investigation of bovine serum albumin (BSA) attachment onto self-assembled monolayers (SAMs) using combinatorial quartz crystal microbalance with dissipation (QCM-D) and spectroscopic ellipsometry (SE)	The loosely interacting layer is discussed for the adsorption of BSA on the neutral surface compared with the charged hydrophobic and/or hydrophilic surfaces.	10.1371/journal.pone.0141282
15	2017	Characterizing protein–protein interaction in high-concentration monoclonal antibody systems with the quartz crystal microbalance	The reversible layer and associated changes in signal are described for high protein concentrations and the viscoelastic behaviors associated with the layer.	10.1039/c7 cP05711c
16	2020	Impacts of carrier properties, environmental conditions, and extracellular polymeric substances on biofilm formation of sieved fine particles from activated sludge	The loosely interacting layer is described for determining the effect of extracellular polymer substances (EPS) on microbial adhesion and biofilm formation with the loosely bound EPS being more conducive to microbial attachment than the tightly attached EPS.	10.1016/j.scitotenv.2020.139196
17	2022	Dissolution mechanism of supported phospholipid bilayer in the presence of amphiphilic drug investigated by neutron reflectometry and quartz crystal microbalance with dissipation monitoring	The loosely interacting layer is mentioned in the context of forming a viscoelastic layer on the dissolution of a phospholipid bilayer with approximately 200 mM amitriptyline hydrochloride, an ionizable amphiphilic drug.	10.1016/j.bbamem.2022.183976
18	2023	Probing the adjustments of macromolecules during their surface adsorption	The loosely interacting layer is discussed with respect to the adsorption of BSA on grafted polymer and bare gold surfaces.	10.1021/acsami.5b01138
19	2023	Scalable fabrication of reversible antifouling block copolymer coatings via adsorption strategies	The loosely interacting layer is indicated regarding studying the antifouling behavior of polymer-based coatings by combining the adsorption strategies from complex coacervate core micelles (C3Ms) and zipper brush method on hydrophobic surfaces.	10.1021/acsami.3c01060

The first part of this study tests the hypothesis that the Δ*f* associated with the loosely interacting macromolecules as obtained by QCM-D acts as a novel self-association metric that corroborates colloidal stability indicators in aggregation studies. The second part of the study focuses on testing the use of the novel QCM-D metric (Δ*f*3 associated with the loosely interacting layer) for assessing the self-association of mAbs and their solution behavior. Furthermore, the potential of the novel metrics for predicting the solution behavior of mAbs at high concentrations is demonstrated, and its predictive power is tested against kD-DLS, the most frequently used method of assessing self-association. Also, the reliability and robustness of this new metric are compared to those of the kD-DLS method. Herein, we show that the approach of specifically using the loosely interacting or reversibly bound layer at the solid surface is novel, reliable, and versatile, and the use of QCM-D along with orthogonal techniques would potentially present a promising strategy to characterize the self-association of mAbs underlying high-concentration solution behavior among other properties.

## 2 Experimental Section

### 2.1 Materials

BSA (lyophilized powder, essentially globulin free ≥99%), l-histidine monohydrochloride monohydrate, and l-histidine were obtained from Sigma-Aldrich Co. (St. Louis, MO). Sodium acetate and acetic acid were obtained from Fisher bioreagents (Pittsburgh, PA), and both monobasic and dibasic potassium phosphate buffer components were purchased from EMD Millipore (Burlington, MA). All of the chemicals obtained were of reagent grade. Several mAbs, including tocilizumab, omalizumab, fremanezumab, dupilumab, cetuximab, trastuzumab, and evolocumab, were extracted from commercially available therapeutic products, and anrukinzumab was acquired from Creative Biolabs (Shirley, NY).

### 2.2 Preparation of mAbs and BSA

The extraction and purification of mAbs from their commercialized product were performed by surfactant removal following the buffer exchange in 10 mM histidine buffer, pH 6.0. Surfactant was removed from the drug product formulations employing DetergentOUT Tween spin columns (G-Biosciences; St. Louis, MO), and buffer was exchanged using Vivaspin ultrafiltration spin column with a 30 kDa molecular weight cutoff (MWCO) membrane (Sartorius, Stonehouse, U.K.). Solutions of mAbs at a concentration of 1–10 mg mL^–1^ were prepared after filtering the buffer exchanged samples using filters of pore size 0.2 μm with sterile PES syringe filter systems (Cytiva, Marlborough, MA).

For viscosity and opalescence measurements, the solutions of tocilizumab and omalizumab were concentrated using Sartorius centrifugal concentrators to concentrations of 150 mg mL^–1^ and 80 mg mL^–1^, respectively. The samples were then diluted to 20, 40, 60, 80, 100, and 120 mg mL^–1^ for tocilizumab, and to concentrations of 20, 40, and 60 mg mL^–1^ for omalizumab. The concentrations were cross-checked by spectrophotometric measurements employing a NanoDrop 2000 (ThermoScientific, Waltham, MA).

BSA was dissolved in 10 mM histidine, acetate, and phosphate buffers, with pHs of 6, 4.5, and 7.4, respectively, to prepare a stock solution of 20 mg mL^–1^ and filter. The BSA solution with 10 mg mL^–1^ was diluted from the stock solution for the Flow Imaging Microscopy study. The concentration of BSA was checked by using a NanoDrop 2000 at 280 nm.

### 2.3 Quartz Crystal Microbalance with Dissipation

QCM-D measurements were performed using a Qsense Explorer (Biolin Scientific, Gothenburg, Sweden) and a single flow chamber with a peristaltic pump (Ismatec, Grevenbroich, Germany) at a flow rate of 10 or 150 μL min^–1^. The temperature was maintained at 20 °C during the experiments by using the internal control system of QCM-D. Gold sensors (QSX 301) were obtained from Biolin Scientific and used to study protein adsorption. Before the experiments, the sensors were cleaned using UV/O3 treatment, followed by a chemical treatment in which sensors were immersed in H_2_O/ammonia/hydrogen peroxide with a ratio of 5:1:1. The sensors were then rinsed with ultrapure water and cleaned again using UV/O3 treatment. The sensors were eventually washed with ultrapure water and dried under filtered air. The frequency shifts Δ*f* and dissipation changes Δ*D* were measured at 6 overtones, i.e., *i* = 3, 5, 7, 9, 11, 13. The third overtone was the main frequency used herein, as all other overtones gave qualitatively similar results.

QCM-D experiment consisted of a series of sequential steps of exposure and wash: (a) A reference baseline was established using the 10 mM histidine buffer, pH 6.0, unless specified otherwise. The baseline was considered stable when the values for Δ*f*, and Δ*D* did not drift more than 1 Hz and 0.2 × 10^–6^ per 10 min, respectively. (b) Next, the protein solution was introduced into the flow cell and allowed to run to achieve maximum saturation until the frequency and dissipation values reached a steady state. (c) Then, the surface was rinsed with baseline buffer until a stable plateau was reached to remove any loosely bound protein. (d) Finally, the fluid path was primed with Milli-Q water for 30 min to clean the system, followed by extensive flushing with sodium dodecyl sulfate (SDS) solution for the next 30 min. To finish cleaning the fluid path, it was rinsed again with water for 25 min and dried with dust-free air. The Δ*f* of the loosely interacting layer was calculated by subtracting the Δ*f* of the total adsorbed protein layer at the steady state obtained in step (b) and Δ*f* of the irreversibly adsorbed layer obtained from the rinsing step (c). In order to obtain information about the thickness and mechanical properties of the adsorbed layer, the data from all overtones were modeled using the Smart Fit function of the Dfind software.

For the adsorption of BSA in different formulations, QCM-D was conducted in a single and separate experiment; a concentration of 20.0 mg mL^–1^ of BSA was used and allowed to flow in different formulations, namely, acetate, histidine, and phosphate buffers with pHs of 4.5, 6.0, and 7.4, respectively. The flow rate used for this experiment was 150 μL min^–1^. The experiment was initiated with baseline stabilization in a 10 mM acetate buffer at pH 4.5. The adsorption of BSA followed this in the same acetate buffer and subsequently in 10 mM histidine buffer at pH 6.0. Finally, there was another adsorption in a 10 mM phosphate buffer at pH 7.4 in the same experimental run.

To conduct QCM-D experiments employing mAb samples, the flow rate of 10 μL min^–1^ was utilized, which allows for reaching the saturation using smaller amounts of sample. In an experiment in which omalizumab and tocilizumab were used as representatives of poorly and well-behaved mAb, respectively, the concentration range of 1, 2, 5, and 10 mg mL^–1^ was used. In the other experiments where a set of eight mAbs was used, a concentration of 10 mg mL^–1^ was employed. All measurements were performed 3 times using separately prepared samples and averages and standard deviations were calculated and presented.

### 2.4 Viscosity Measurements

Viscosity measurements were performed at 20 °C employing m-VROC (RheoSense, San Ramon, CA) and processed using the instrument’s control software. The rheometer is based on measuring an accurate pressure drop employing an array of pressure sensors of the microfluidic chip. Viscosity measurements were performed for varying concentrations of tocilizumab (1, 2, 5,10, 20, 40, 60, 80, 100, and 120 mg mL^–1^) and omalizumab (1, 2, 5, 10, 20, 40, 60, and 80, mg mL^–1^). Each concentration was tested at different shear rates (100, 200, 500, 1000, 2000, and 3000 1/s). The measurement at higher shear rates was impossible for some higher concentrations due to pressure increase beyond the capacity of the system, and therefore, the results of the lower shear rates were reported. It is noteworthy that the viscosity values (reported in centipoise [cP]) were not shear rate dependent in the measured range. For the measurements, the A05 chip with ID 22RA05100657 was used. 300 μL of the samples was filled in the glass syringe, and three measurements were performed for each concentration. Out of 300 μL, 100 μL was used for each measurement, the first of which was ignored to minimize the potential errors associated with imperfect initial filling of the sensor cell. The accurate viscosity determinations were considered based on the “Min Slope Fit Rsqrd” values above 0.98 and % full-scale value between 5 and 95% using shear rates 100, 200, 500, 1000, 2000, and 3000 s^–1^.

### 2.5 Flow Imaging Microscopy

The particle size distribution of micrometer-sized particles was measured using an 8000 series FlowCam (Yokogawa Fluid Imaging Technologies, Scarborough, Maine). The measurement was conducted with a 10× objective lens, an FOV80 flow cell, and a sample volume of 300 μL at a flow rate of 150 μL min^–1^. Samples were captured at 5 μm to the nearest neighbor and imaged using thresholding parameters of 20 for both dark and light pixels at a rate of 20 frames per second. The instrument was autofocused using the NIST 15 μm polystyrene bead standard (Duke Standards, Fremont, CA) before measuring the samples. The flow cell was flushed between the measurements with ultrapure water, SDS 1%, and an alkaline-based reagent to warrant a clean flow cell. The number and size of the subvisible particles in the range of 1–100 μm were determined and plotted per size categories (>1, >2, >5, and >10 μm) in different formulations.

To assess the colloidal stability of BSA formulations, BSA samples were prepared at a concentration of 10 mg mL^–1^ in acetate, histidine, and phosphate buffers with pH values of 4.5, 6.0, and 7.4, respectively. The samples were then incubated at 5 °C for 5 days. After the incubation period, duplicate samples were taken and analyzed for subvisible particles using flow imaging microscopy.

### 2.6 Opalescence Measurements

The experiment involved measuring the light scattering signal of tocilizumab solutions (with concentrations of 10, 20, 40, 60, 80, 100, 120, and 150 mg mL^–1^) and omalizumab solutions (with concentrations of 20, 40, 60, and 80 mg mL^–1^) using a SpectraMax M5 spectrometer (Molecular Devices, San Jose, CA). To perform this, 200 μL of each sample was added in triplicate to a Corning 96-well clear UV-transparent flat-bottom microplate (Corning, Inc., New York, NY) and measured in the 340–360 nm range. The baseline subtraction with a histidine buffer was performed for each measurement. A measure of the opalescence of mAb samples was obtained by using the mean optical density with 5 nm increments, which was calculated as the average of three separate sets of measurements.

### 2.7 Diffusion Interaction Parameter by DLS

Dynamic light scattering studies were performed in a 96-microwell glass-bottom sensoplate microplate (Greiner Bio-One, Monroe, NC) using a DynaPro Plate Reader II (Wyatt, Santa Barbara, CA). The stock solution of mAbs, i.e., 10 mg mL^–1^, was diluted to a concentration range of 1, 1.5, 2, 2.5, 3, 3.5, 4, 4.5, and 5 mg mL^–1^ concentrations in 10 mM histidine buffer, pH 6.0. For the loading of samples, 80 μL of samples were added in triplicates for each mAb concentration. Next, 10 acquisitions were measured for a duration of 5s at 20 °C using an autoattenuated laser wavelength of 825 nm. Dynamics software version 7.8 (Wyatt Technology, Santa Barbara, CA) for fitting the data with a cumulant model was utilized for data analysis. The kD-DLS representing an average of three measurements for each mAb was calculated in units of mL g^–1^ as a ratio of linear regression slope to the intercept from the plot of diffusion coefficient versus the concentration of mAbs.

### 2.8 Robustness Studies

For robustness studies, the effect of impurities and temperature variation on the mAb samples was evaluated. For determining the effect on trastuzumab, samples with concentrations from 1 to 5 mg mL^–1^ were spiked with 1 μm polystyrene microspheres to reach a final concentration of 2.0 million particles mL^–1^. Next, a set of three measurements were conducted at 20 °C to analyze the trastuzumab sample using QCM-D and DLS techniques. The loosely interacting layer was evaluated using Δ*f* in QCM-D, while the kD-DLS value was determined by DLS through linear regression with the trastuzumab concentration as an independent variable. The average Δ*f* and kD-DLS values and their standard deviations from three separate measurements using separately prepared samples were calculated and are presented.

DLS studies were carried out to investigate the effect of temperature on kD-DLS as kD measurements require several DLS measurements at different concentrations of the protein, and therefore, the risk of having slightly different temperatures in each measurement is high. The study examined the effect of temperature (seven temperatures 20.0 to 26.0 °C) on the diffusion coefficient of tocilizumab at all concentrations ranging from 1 to 5.0 mg mL^–1^. Studies were performed for three sets of separately prepared samples, and the kD-DLS value was determined by fitting a linear regression as described above.

### 2.9 Comparative Statistical Analysis

To compare the novel metrics of the loosely interacting layer with kD-DLS, we calculated Δf for the loosely interacting layer of all eight mAbs at a concentration of 10 mg mL^–1^ employing QCM-D. We then correlated this value with kD-DLS for the mAbs used in the concentration range of 1–5 mg mL^–1^. The average of three measurements using separately prepared samples was taken for each mAb by both methods. To evaluate the rank order correlation between Δ*f* and kD-DLS, Spearman’s correlation coefficient was utilized. We also calculated the Pearson correlation coefficient for comparing Δ*f* and Δ*D* with the kD-DLS values. Additionally, the average relative error for both metrics was calculated to determine the reliability of the metrics.

## 3 Results and Discussion

### 3.1 Assessment of Protein Self-Association and Colloidal Stability in Different Formulations

**Figure 2:**
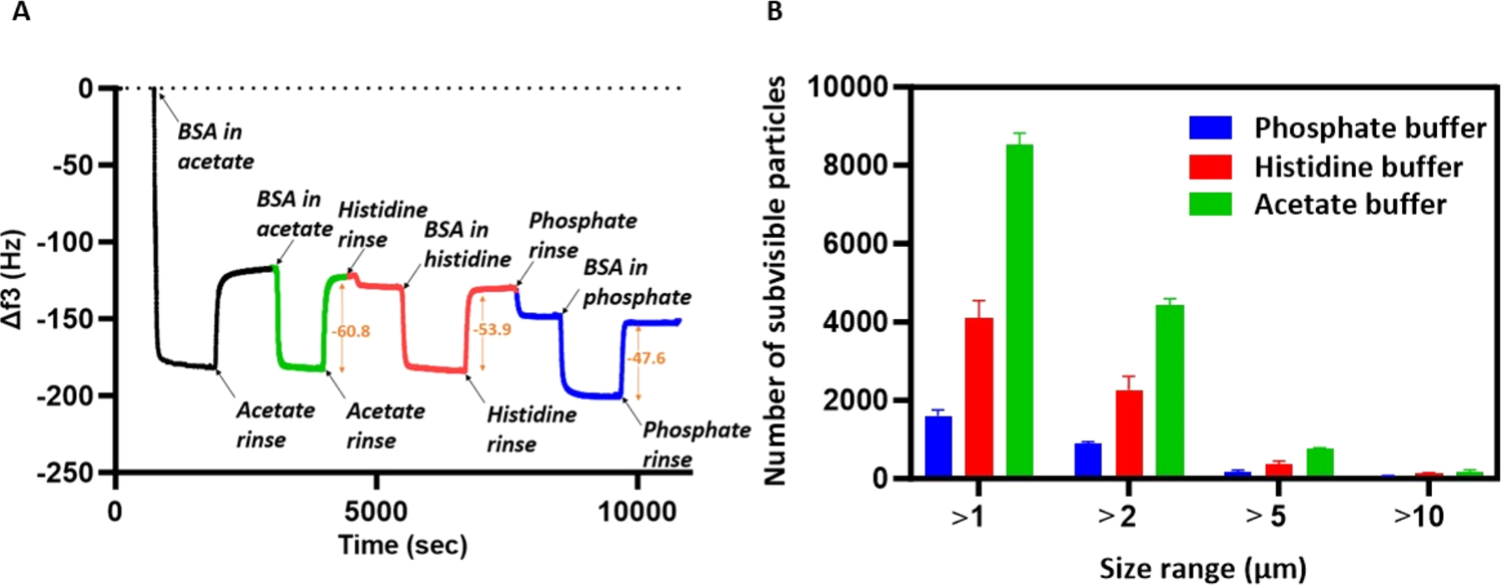
Assessment of BSA self-association and colloidal stability in different formulations. (A) Frequency shifts due to the adsorption of BSA from three different formulations in 10 mM histidine, acetate, and phosphate buffers, with pHs of 6, 4.5, and 7.4, respectively (Δ*f*_acetate_ > Δ*f*_histidine_ > Δ*f*_phosphate_). (B) The number of subvisible particles detected in formulations after storage. The bars show the average and range from two separate samples for different aggregate size ranges (larger than 1, 2, 5, and 10 μm).

In order to test the basic relations of the self-association metric from the loosely interacting layer with colloidal stability of protein formulations, we conducted experiments to measure the Δ*f* associated with the loosely interacting layer for bovine serum albumin (BSA), in different buffers (acetate, histidine, and phosphate buffers) with pH values of 4.5, 6, and 7.4, respectively. BSA was used as a model protein in this proof-of-concept study to assess self-association by performing adsorption studies for the protein in different formulations in a single run. Although BSA is not a therapeutic protein, these studies would help demonstrate the potentials of the method and its applicability to other proteins and various buffer conditions. In the initial experiment, a single run was performed which started with the flow of BSA in acetate formulation to achieve a baseline coating of the surface with BSA. Subsequently, another cycle of BSA in acetate and a rinse with acetate buffer was performed resulting in the largest loosely interacting layer (Δ*f =* – 60 Hz) of BSA (Figure 2A). At this stage, Δ*f* associated with the irreversibly adsorbed layer remained unchanged. Upon replacement of the buffer with histidine buffer, a shift in the Δ*f* of the irreversibly adsorbed layer was observed that had to do with the extent of swelling of the adsorbed layer in the buffer. The following run of BSA in histidine formulation and rinse with a histidine buffer resulted in the return of the signal to the baseline in histidine and revealed an intermediate loosely interacting layer in histidine (Δ*f =* – 53.9 Hz). Finally, and similarly, a later run involving the flow of phosphate buffer, followed by a BSA in phosphate formulation and rinse with a phosphate buffer exhibited the smallest loosely interacting layer of BSA (Δ*f =* – 47.6 Hz). Clearly, the Δ*f* values of the loosely interacting layer were highest in acetate formulation which was also confirmed when separate experiments were performed for each buffer (Figure 2A and Supporting Figure 1). Studying the interaction of molecules in one experiment versus separate experiments may provide advantages in terms of the usage of proteins and the duration of the experiments. Considering that the irreversibly adsorbed amount may be influenced by the type of buffer, the experiments were initiated with BSA in acetate buffer, which gives the largest adsorbed layer. In addition, an assessment of the kD-DLS values for the BSA in three formulations revealed that the lowest kD-DLS was associated with acetate buffer in line with Δ*f* values that suggest the highest self-association for this buffer (Supporting Figure 2). Colloidal stability studies were performed by storing the BSA in three buffers at 5 °C for 5 days, following which the number of subvisible particles in the micrometer size range was quantified by flow imaging microscopy (Figure 2B). BSA prepared in acetate formulation formed the most subvisible particles, while the least number of subvisible particles was found in the phosphate formulation, in line with the Δ*f* values from QCM-D. This correlation indicates the potential of the self-association metric obtained by QCM-D in assessing interactions between molecules and predicting the colloidal stability of the system. The observation can be attributed to the fact that BSA, with its isoelectric point (pI) around 4.7,^39^ is uncharged when formulated in acetate buffer and experiences no major electrostatic repulsion between its molecules, promoting self-association. It has to be realized that aggregation is influenced by multiple factors including conformational stability, interfacial, and other stresses as well as colloidal stability which are all in play in a real-world scenario, and therefore, this method may not be able to predict the aggregation behavior in complex systems. Defante et al.^40^ discussed the secondary layer in an adsorption study and mentioned that this layer was not predictive of the formation of subvisible particles.

### 3.2 Prediction of Solution Behavior of mAbs through Application of QCM-D Metrics

**Figure 3:**
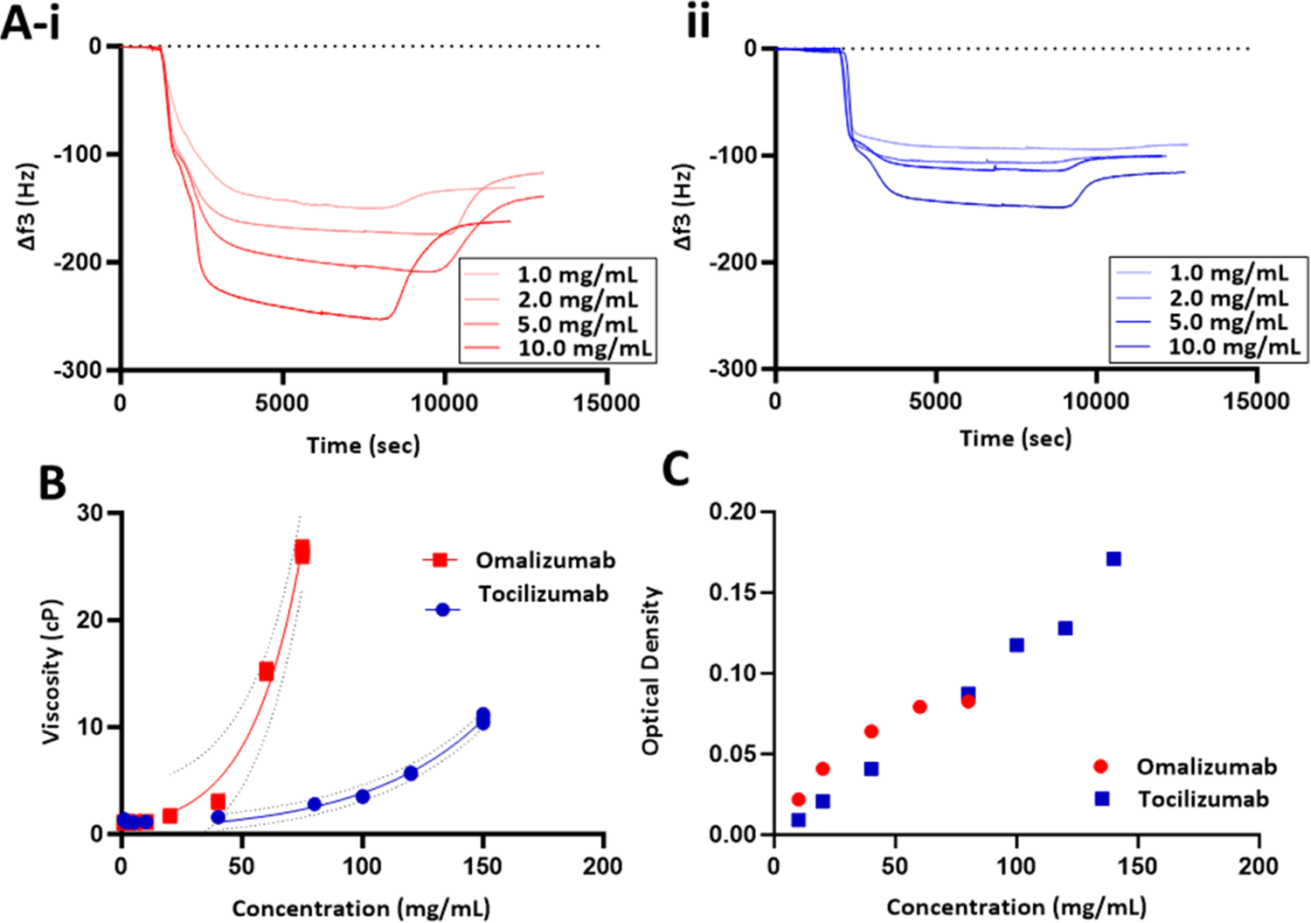
Prediction of the solution behavior of mAbs through the application of QCM-D metrics. (A-i) QCM-D profile depicting the changes in frequency (*f*3) overtones for a range of concentrations, i.e., 10, 5, 2, and 1 mg/mL for omalizumab. (A-ii) QCM-D profile depicting the changes in frequency (*f*3) overtones for a range of concentrations, i.e., 10, 5, 2, and 1 mg/mL for tocilizumab. (B) Viscosity measurements for omalizumab and tocilizumab for a concentration range from 40 to 150 mg/mL. Solid lines show the exponential fit, and dashed lines show the 95% prediction. Note that viscosity does not change up to 10 mg/mL for any of the mAbs. (C) Opalescence measurements for different concentrations of omalizumab and tocilizumab.

To understand the correlation between QCM-D metrics with the solution behavior of mAbs, we conducted a QCM-D experiment for omalizumab and tocilizumab, a poorly^41^ and a well-behaving mAb,^16^ respectively, for a range of concentrations (1–10 mg mL^–1^). Interestingly, in the case of omalizumab, a distinct and large loosely interacting layer was detected for all concentrations (Figure 3A-i, and Supporting Figures 3 and 4A), whereas this layer was absent or considerably smaller in the case of tocilizumab (Figure 3A-ii and Supporting Figures 3 and 4B). At a concentration of 10 mg mL^–1^, the Δ*f* of the loosely interacting layer for omalizumab was −89.62 Hz, while for tocilizumab, it was −28.15 Hz, and similar trends were observed for all frequency overtones and dissipations (Supporting Figure 4). The thickness of the layers obtained from modeling of the data (Supporting Figure 5 and Table 1) corroborates the Δ*f* observations suggesting that dissipation and thickness could also be used as potential metrics for self-association. However, we have selected Δ*f* to be utilized as the primary metric for the purpose of this proof-of-concept study. It is important to realize that the viscosity of tocilizumab and omalizumab formulations were the same across all of the low-concentration samples, i.e., up to 10 mg mL^–1^ while omalizumab showed a much higher viscosity at high-concentration formulations (Figure 3B and Supporting Figure 6). Depending on the type of mAb, at an intermediate concentration (appears to be in the range of 20–40 mg mL^–1^) where the high number of molecules in solution brings them in close proximity to each other, the viscosity of the samples started to increase exponentially. Fitting of the viscosity data points in this range (Figure 3B) revealed that the viscosity value would increase to around 90 cP for 100 mg mL^–1^ omalizumab, whereas it would still be under 4 cP for tocilizumab, making the former a challenging mAb in terms of solution behavior and development of high-concentration formulations.^13^ These data clearly suggest that these novel metrics based on characteristics of the loosely interacting layer at relatively low concentrations can predict the solution behavior of the mAbs. In this regard, Oom et al. examined high-concentration formulations (50 and 100 mg mL^–1^) where the viscosity of the formulation is already significantly high. Previous studies by Hartl et al. and Patel et al. have demonstrated that the viscosity of high-concentration antibody formulations substantially impacts changes in frequency (Δ*f*) from QCM-D. In contrast, our findings indicate that at concentration ranges below 10 mg mL^–1^, where the viscosity of the formulation remains similar or unchanged, the loosely interacting layer is different based on the intrinsic properties of the protein. Furthermore, the optical density of high-concentration formulation was also measured as an indicator of opalescence, and it was comparable for both tocilizumab and omalizumab (Figure 3C) which is in line with previous reports that suggest that high self-association is typically manifested in either high viscosity or high opalescence when concentration increases.

### 3.3 Comparison of QCM-D Metrics with That of kD-DLS for mAbs

**Figure 4:**
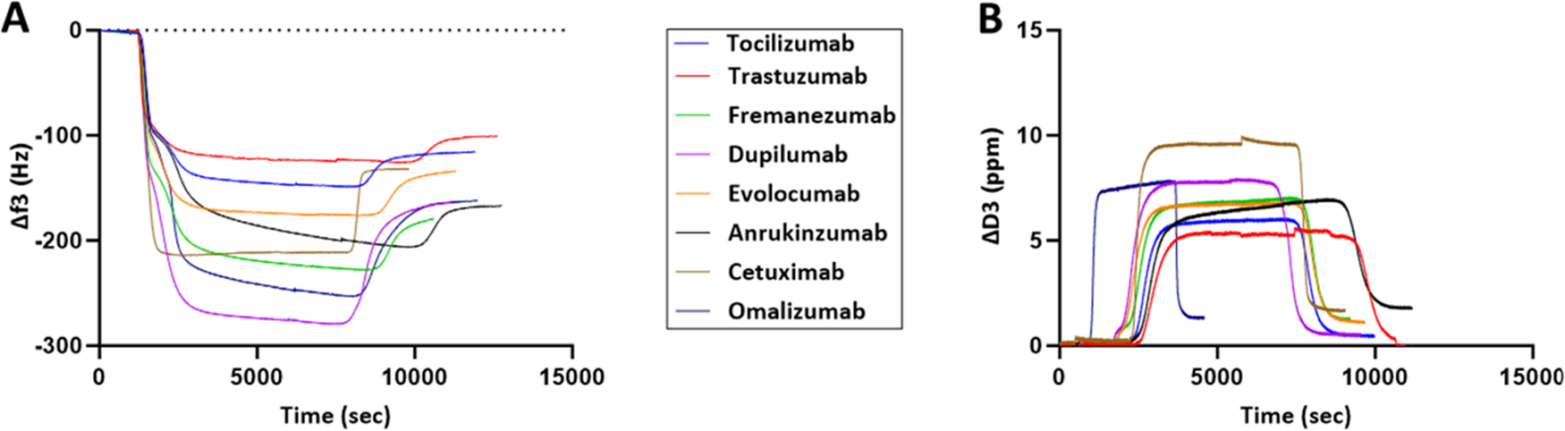
QCM-D metric of the loosely interacting layer for eight mAbs. (A) Frequency plots and (B) dissipation plots for antibodies spanning well and poorly solution behavior.

At the next stage of method development, we sought to compare the novel metrics of the loosely interacting layer with the currently employed kD-DLS for eight mAbs, spanning good, intermediate, and poor solution behavior. We calculated kD-DLS by measuring the diffusion coefficient values as a function of mAbs concentration at 20 °C (Supporting Figure 7). The characteristics of the loosely interacting layer were determined for all mAbs at 10 mg mL^–1^ concentration and terms of Δ*f* and Δ*D* were observed to be correlated (Figure 4 and Supporting Figure 8). Most importantly, a strong rank order correlation between kD-DLS and Δ*f* for the loosely interacting layer was observed with Spearman’s correlation coefficient of ρ = 0.809 (Table 2, and Supporting Figure 9 and Table 2), indicating a strong positive monotonic relationship between the two methods being compared. The relationship between the two methods is statistically significant, further indicating the parameters of the loosely interacting layer as metrics of self-association (Pearson correlation coefficients have also been presented in Supporting Table 2). It is noteworthy that we used experimental conditions that have been commonly used for kD-DLS measurements (mAb in 10 mM histidine buffer pH 6 without any other excipient), and the strong rank order correlation suggests that the Δ*f* for loosely interacting layer could serve as an alternative to kD-DLS in potentially predicting the solution behavior of the mAb molecules. In this regard, kD-DLS values are independent of the concentration, whereas the Δ*f* values reported herein are associated with the concentration of 10 mg mL^–1^.

**Table 2:** Rank Order Correlation between Δ*f*3 from QCM-D and kD-DLS for Eight mAbs^a^

monoclonal antibodies	QCM-D metric	relative error for QCM-D metric (%)	diffusion interaction parameter; kD-DLS	relative error for kD-DLS (%)	rank (Δ*f*3)	rank (kD-DLS)
Trastuzumab	–23.9 ± 2.7	11.3	51.7 ± 2.5	4.8	1	2
Tocilizumab	–31.7 ± 2.1	6.6	64.9 ± 7.3	11.2	2	1
Evolocumab	–36.9 ± 5.0	13.5	22.8 ± 8.0	35.1	3	4
Anrukinzumab	–40.5 ± 2.1	5.2	7.5 ± 4.0	53.3	4	5
Fremanezumab	–47.6 ±4.3	9.0	36.8 ± 13.6	37.0	5	3
Cetuximab	–79.4 ± 9.4	11.8	–42.4 ± 6.3	14.9	6	8
Omalizumab	–82.4 ± 15.5	18.8	–22.9 ± 1.8	7.9	7	7
Dupilumab	–112.4 ± 4.4	3.9	3.0 ± 13.1	436.7	8	6

a Spearman’s correlation coefficient = 0.809; *P*-value = 0.027.

### 3.4 Evaluation of Robustness of DLS and QCM-D Metrics under Temperature Variability and Impurities

**Figure 5:**
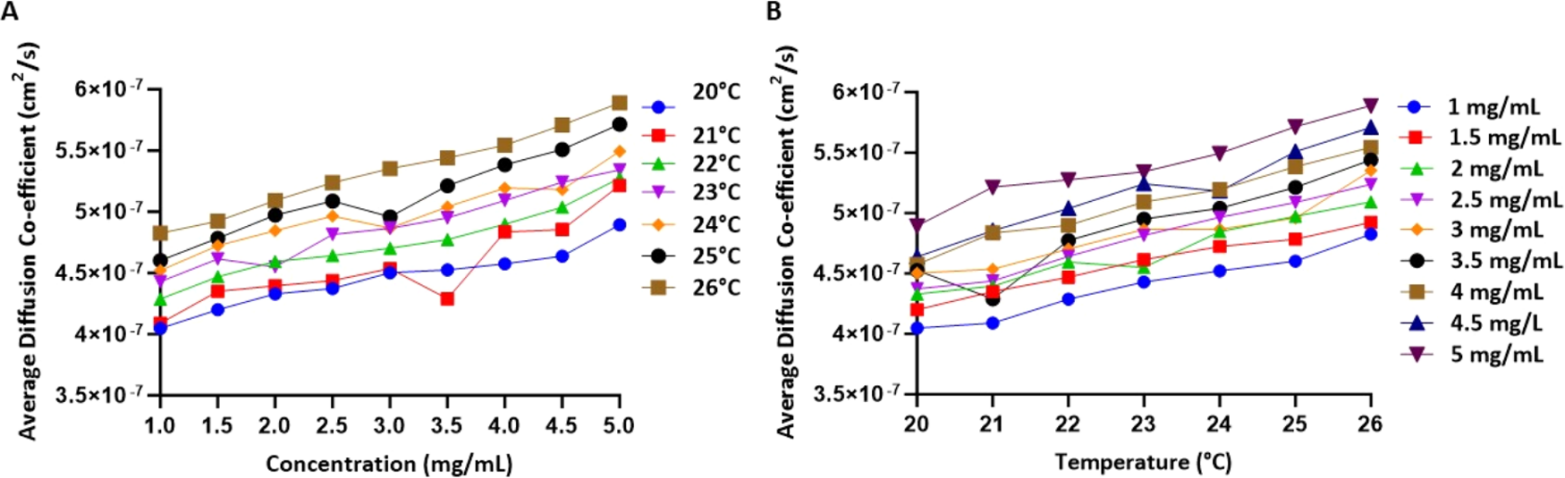
Sensitivity of kD-DLS for changes in temperature and concentration. (A) Average diffusion coefficient vs concentration for a temperature ranging from 20 to 26 °C with a difference of 1 °C. (B) Average diffusion coefficient vs temperature for a concentration ranging from 1 to 5 mg/mL with a 0.5 mg/mL difference.

DLS measurements are highly sensitive to the presence of impurities, including protein aggregates, which may be common in mAb formulations, particularly for self-associating mAbs, and to variations in temperature. Therefore, in the last part of this study, we performed reliability and robustness studies to effectively compare and evaluate the QCM-D-based method with kD-DLS. We investigated the effect of temperature by measuring the diffusion coefficient in the range of 20–26 °C at mAb concentrations employed for kD-DLS measurements (1–5 mg mL^–1^). These studies indicated that the average change in diffusion coefficient for variation in temperature by 1 °C was 1.40 × 10^–8^ cm^2^ s^–1^ as compared to an average of 5.76 × 10^–9^ cm^2^ s^–1^ for 0.5 mg mL^–1^ of mAb concentration (Figure 5). These results confirmed that kD-DLS measurements are sensitive to temperature variation. Sensitivity to temperature is particularly important because kD-DLS calculation requires multiple measurements at several concentrations. In this regard, plate-based DLS systems such as the one employed in this study provide a more efficient and uniform temperature control for kD-DLS assessment. Moreover, the QCM-D metric is measured in a single measurement in a well-controlled flow chamber with minimal temperature variations (Supporting Figure 10 shows the actual temperature for a typical QCM-D experiment).

**Figure 6:**
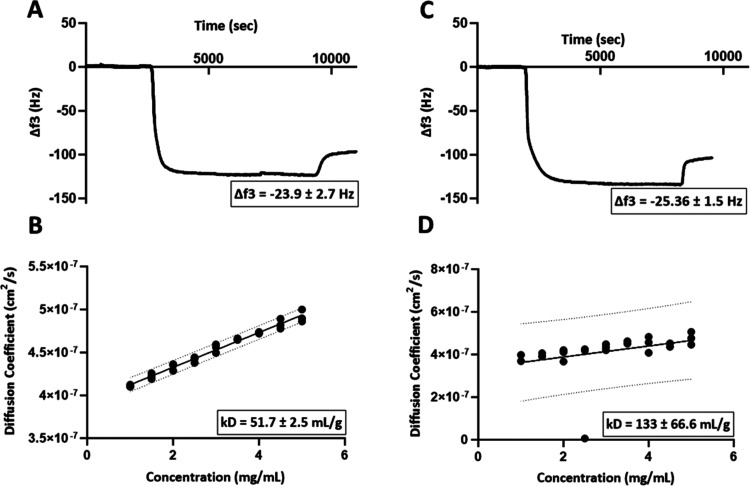
Effect of robustness by spiking the samples with polystyrene beads and its comparison with the control by both QCM-D and kD-DLS methods. (A) Control QCM-D experiment, (B) control DLS experiment, (C) spiked QCM-D experiment, (D) spiked DLS experiment. The presence of particles (impurities) did not influence the QCM-D measurement of the self-association metric whereas the kD-DLS measurements were greatly affected.

Next, to mimic the potential presence of particulate impurities such as aggregates and external impurities like dust in samples, 1 μm polystyrene microspheres were spiked into the mAb solutions, and their effect on kD-DLS and Δ*f* measurements was evaluated. It was found that the presence of impurities did not affect the Δ*f* values of the loosely interacting layer while it significantly changed the kD-DLS value and made it difficult to obtain reliable diffusion coefficient values as shown by the broadened 95% confidence interval prediction bands (Figure 6). Therefore, the characterization of self-associating mAbs poses significant challenges due to their inherent propensity for aggregation, which can render conventional techniques, such as DLS unreliable. The wide interval associated with the shift in slope, although indicative of self-association, presents a problematic scenario, necessitating further investigation and refinement of the analytical measurements for self-associating mAbs. It is noteworthy that in QCM-D measurements, while the presence of particles may influence the total frequency shift observed before or after rinsing, the loosely interacting layer remains unaffected, with the Δ*f* of the loosely interacting layer staying constant. This observation from the spiked polystyrene bead experiment underscores the robustness of the loosely interacting layer as a metric for self-association, even in the presence of impurities and/or aggregates.

In conclusion, we developed a versatile method to directly measure protein self-association under relevant conditions and showed its use in predicting important attributes in mAb development and formulation. Overall, we have for the first time demonstrated that (a) parameters of the loosely interacting layer can be used as metrics of protein self-association and that these metrics may be used to predict the colloidal stability of protein systems as well as solution behavior of mAbs, and (b) these parameters provide metrics that are an alternative to the kD-DLS values that are currently used for measurement of weak interactions and assessment of self-association. This work is significant because it increases the potential to identify the candidates in drug development with weak solution behavior and susceptibility to aggregation which can lead to enhanced efficiency in the development of much needed mAb therapeutics, which, in turn, would free resources for addressing more of the unmet medical needs. The development of a method to directly measure protein self-association would be very beneficial to the scientific community, including drug scale-up capabilities, extending the shelf life of protein drugs, decreasing raw material waste, and improving time to market. Streamlining the drug development process will also benefit the general public by decreasing costs associated with new drug investigations/scale-ups. Furthermore, this novel method for using QCM-D will also find applications in other biomedical fields, such as nanoparticulate delivery systems and biosensing. Thus, this work would provide a foundation for future implementation into therapeutic discovery and development pipelines.

## Data Availability

The data that support the findings of this study are available from the corresponding author upon reasonable request.

## Supporting Information Available

Frequency shifts for loosely interacting layer of BSA prepared in different formulations; plot of diffusion coefficient vs concentration range for BSA in different formulations; frequency changes associated with the loosely interacting layer versus the concentration for omalizumab and tocilizumab; frequency and dissipation plots for all of the overtones for adsorption of omalizumab and tocilizumab; thickness plots obtained from the Dfind Smartfit modeling in QSense Dfind software for adsorption of omalizumab and tocilizumab; thickness values of the different layers measured during a QCM-D run for omalizumab and tocilizumab; table depicting the viscosity measurements for omalizumab and tocilizumab for 40.0 mg/mL concentration as obtained from RheoSense m-VROC, plot of diffusion coefficient vs concentration range for all eight antibodies studied; correlation of frequency change associated with the loosely interacting layer with dissipation change for all eight antibodies studied; frequency changes associated with the loosely interacting layer versus the diffusion interaction parameter kD-DLS for all eight antibodies studied; summary of the statistical analysis of correlations between Δ*f*3 and kD-DLS as well as Δ*D*3 and kD-DLS calculated for all eight mAbs studied; and temperature variability during a representative QCM-D measurement (PDF)

## Notes

R.N. is a member of the scientific advisory board at Coriolis Pharma, Martinsried, Germany.
