# Supplementary material for: Prediction of Self-Association and Solution Behavior of Monoclonal Antibodies Using the QCM-D Metric of Loosely Interacting Layer
Source: Mol Pharm. 2024 Nov 29;22(4):1804–15. doi: 10.1021/acs.molpharmaceut.4c00656 (PMC11979879; doi:10.1021/acs.molpharmaceut.4c00656)
Supplement: Supplementary file 1 — mp4c00656_si_001.pdf [file mp4c00656_si_001.pdf]

Prediction of Self-Association and Solution Behavior of Monoclonal Antibodies Using the QCM-D Metric of Loosely Interacting Layer

Authors: Yusra Rahman[a+], Siddhanth Hejmady[a+] and Reza Nejadnik[a*]

[a] Department of Pharmaceutical Sciences & Experimental Therapeutics, College of Pharmacy, University of Iowa, Iowa City, IA 52242, United States

[+] authors contributed equally.

[*] Corresponding author: reza-nejadnik@uiowa.edu

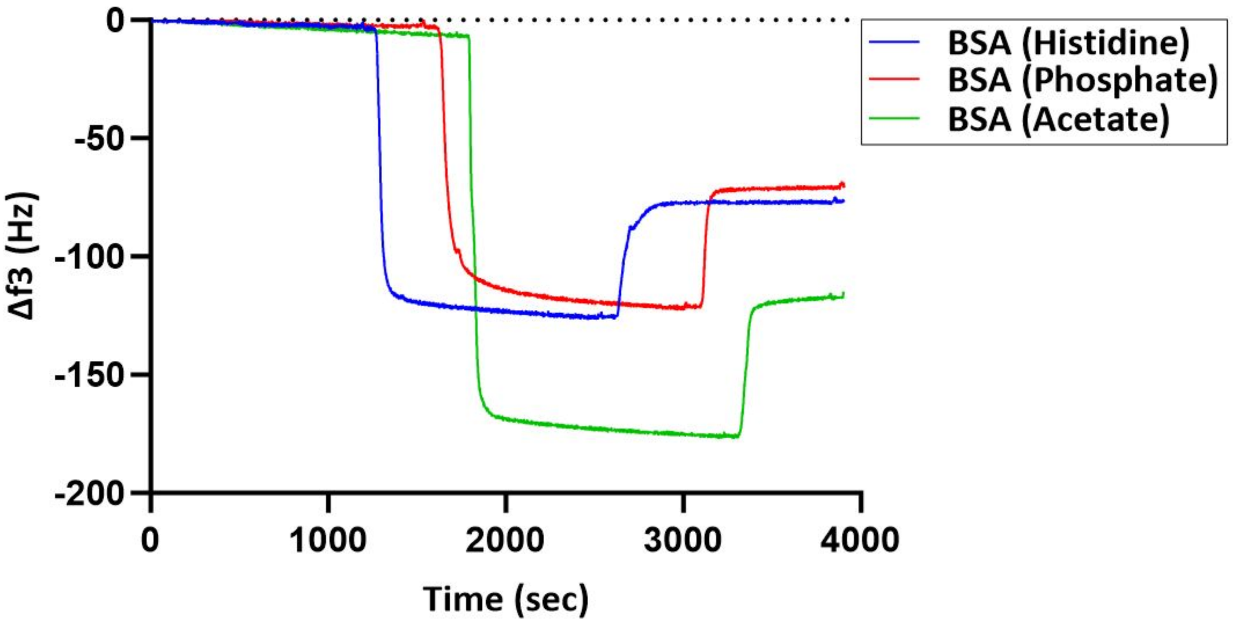

Supplementary Figure 1. Frequency shifts for loosely interacting layer of BSA prepared in different formulations with Δf = -59 Hz in 10 mM, pH 4.5, acetate buffer, Δf = -48.6 Hz in 10 mM, pH 6, histidine buffer, and Δf = -51.07 in 10 mM, pH 7.4, phosphate buffer

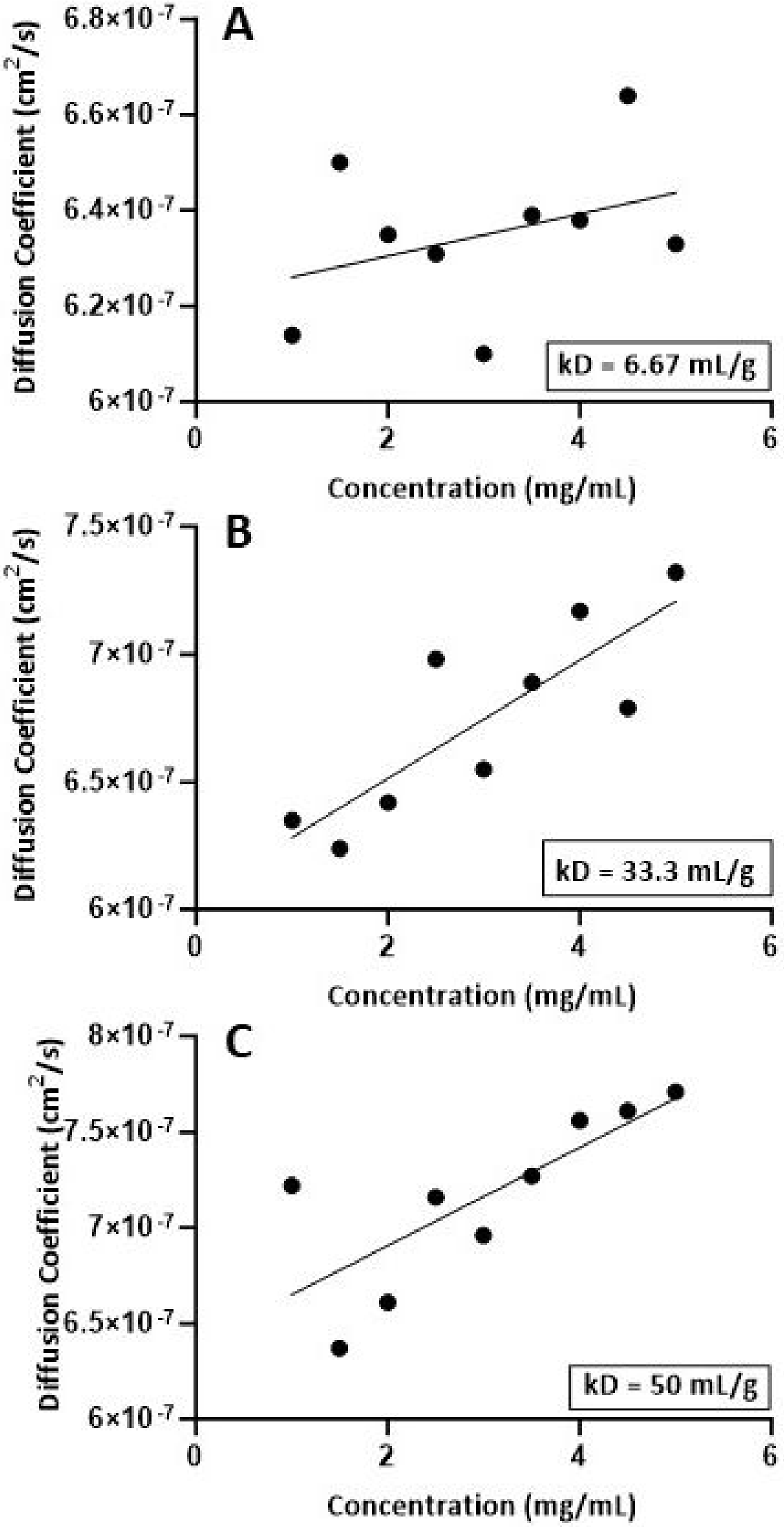

Supplementary Figure 2. A plot of Diffusion Coefficient vs Concentration range (1-5 mg/mL) for BSA in A) 10 mM, pH 4.5, acetate buffer B) 10 mM, pH 6, histidine buffer, and C) 10 mM, pH 7.4, phosphate buffer depicting the diffusion interaction parameter, which is calculated as a ratio of slope and intercept

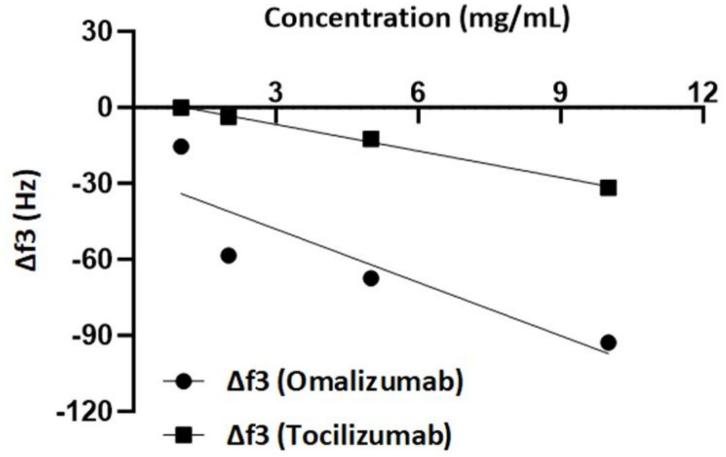

Supplementary Figure 3. Frequency changes associated with the loosely interacting layer, Δ*f* versus the concentration for omalizumab and tocilizumab

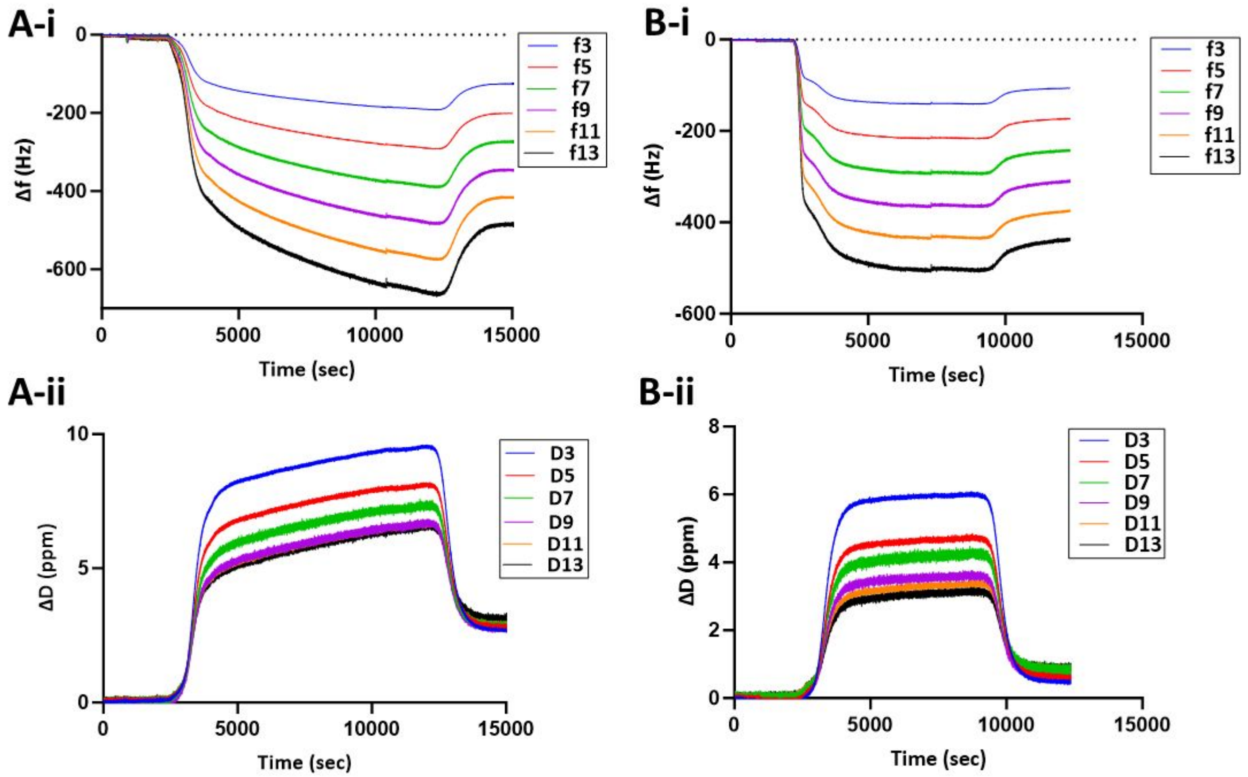

Supplementary Figure 4. Frequency plots for all the overtones for adsorption from a 10 mg mL^{-1} solution of A-i) omalizumab and B-i) tocilizumab. Dissipation plots for all the overtones for A-ii) omalizumab and B-ii) tocilizumab

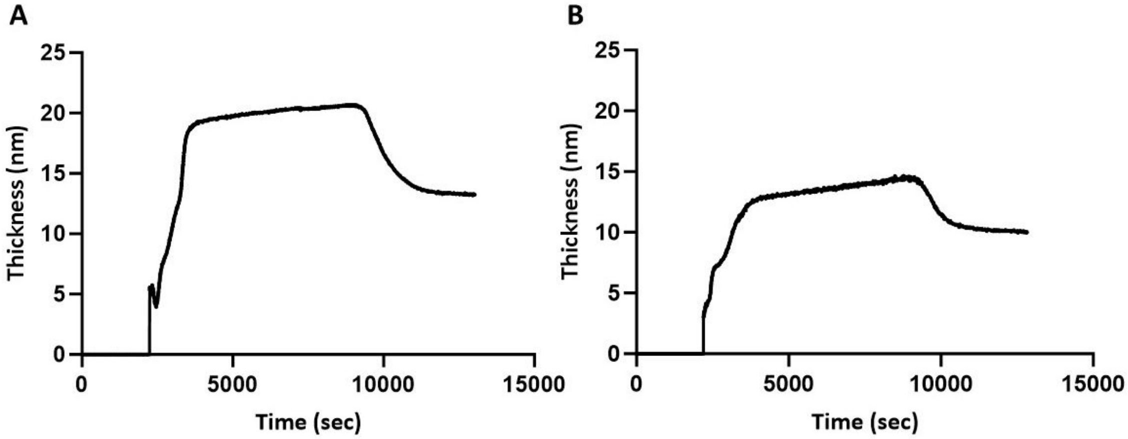

Supplementary Figure 5. Thickness plots obtained from the Dfind Smartfit modeling in QSense Dfind software for adsorption from a 10 mg mL^{-1} solution of A) omalizumab and B) tocilizumab (Also, the associated viscoelastic properties of the loosely interacting layer as predicted by modeling were similar for omalizumab and tocilizumab, although the thicknesses of the layers differ between the two mAbs.)

Supplementary Table 1. Thickness values of the different layers measured during a QCM-D run for omalizumab and tocilizumab (Averages and standard deviations were calculated from data obtained from three separate experiments.)

	**Omalizumab**	**Tocilizumab**
**Thickness (nm)**	**Average ± Standard Deviation**	**Average ± Standard Deviation**
T (before rinse)	22.7 ± 3.9	13.8 ± 0.6
T (after rinse)	15.1 ± 3.8	8.8 ± 1.9
T (loosely interacting layer)	7.5 ± 0.2	5.0 ± 2.0

Segment	Temp, C	Press Drop, Pa/mm	Flow Rate, ul/min	Shear Stress, Pa	% Full Scale	Apparent Shear Rate, 1/s	Apparent Visc, mPa-s	True Shear Rate, 1/s	True Visc, mPa-s	Slope Fit Rsqrd
1	19.98	14	5.4	0.4	1.2	100.1	3.543	100.1	3.543	0.9896
2	19.98	29	10.8	0.7	2.1	200.3	3.579	200.3	3.579	0.9904
3	19.98	63	26.8	1.6	4.9	498.9	3.161	498.9	3.161	0.9952
4	19.98	122	53.7	3.0	9.8	997.7	3.038	997.7	3.038	0.9988
5	19.99	235	107.3	5.9	19.7	1995.4	2.937	1995.4	2.937	0.9994
6	19.99	350	161.0	8.7	29.3	2993.1	2.915	2993.1	2.915	0.9996

Omalizumab 40 mg/mL

Segment	Temp, C	Press Drop, Pa/mm	Flow Rate, ul/min	Shear Stress, Pa	% Full Scale	Apparent Shear Rate, 1/s	Apparent Visc, mPa-s	True Shear Rate, 1/s	True Visc, mPa-s	Slope Fit Rsqrd
1	20.00	6	5.4	0.1	0.4	100.1	1.457	100.1	1.457	0.9853
2	20.00	12	10.8	0.3	0.8	200.3	1.491	200.3	1.491	0.9909
3	20.00	31	26.8	0.8	2.4	498.9	1.556	498.9	1.556	0.9956
4	20.01	64	53.7	1.6	5.1	997.7	1.602	997.7	1.602	0.9987
5	20.00	126	107.3	3.1	10.6	1995.4	1.577	1995.4	1.577	0.9998
6	20.00	188	161.0	4.7	15.8	2993.1	1.564	2993.1	1.564	0.9999

Tocilizumab 40 mg/mL

Supplementary Figure 6. Table depicting the viscosity measurements for omalizumab and tocilizumab for 40.0 mg/ml concentration, as obtained from RheoSense m-VROC (The values for the viscosity were selected based on the manufacturer's instructions, with strict adherence to the criteria of Rsqrd slope > 0.98 and % full-scale value falling between 5% and 95%.)

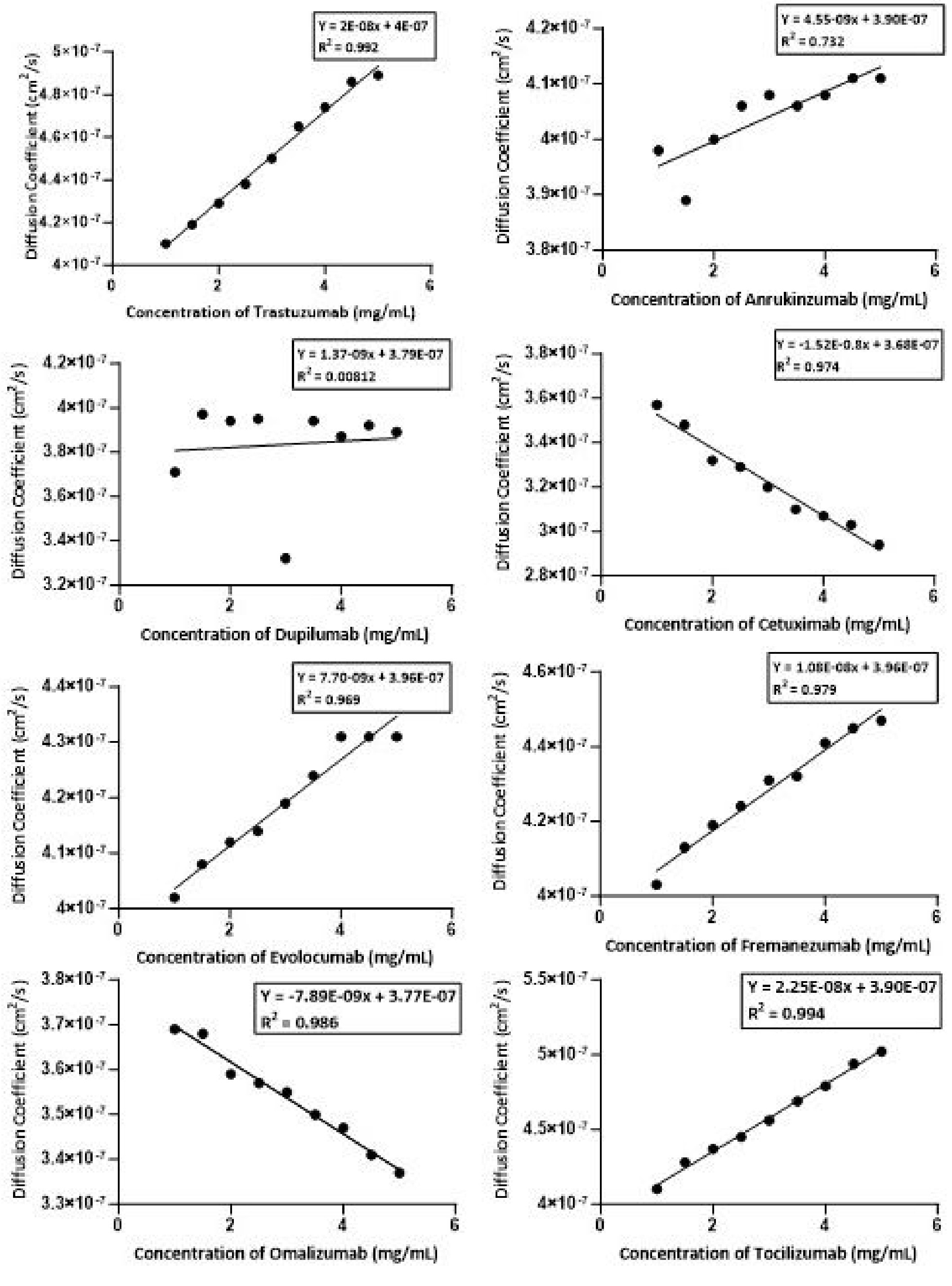

Supplementary Figure 7. A plot of Diffusion Coefficient vs Concentration range (1-5 mg/mL) for all eight antibodies studied

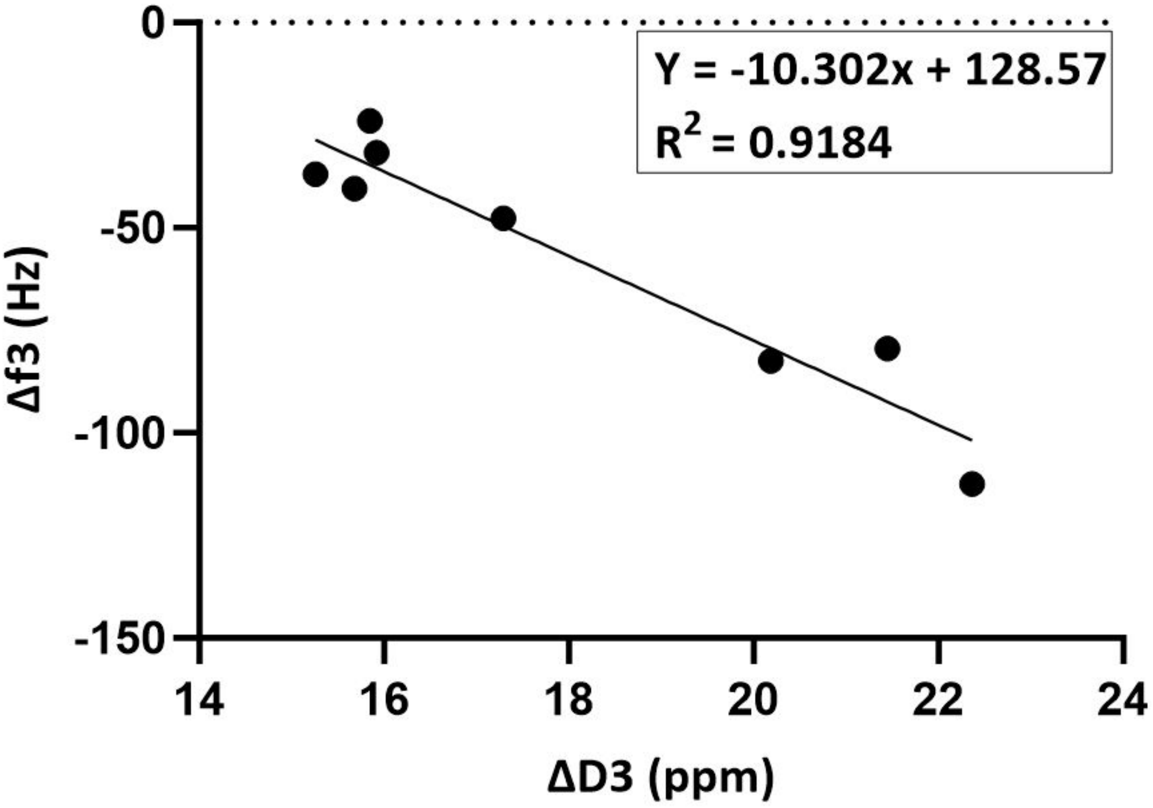

Supplementary Figure 8. Co-relation of frequency change associated with the loosely interacting layer, Δ*f* with dissipation change, Δ*D* for all eight antibodies studied

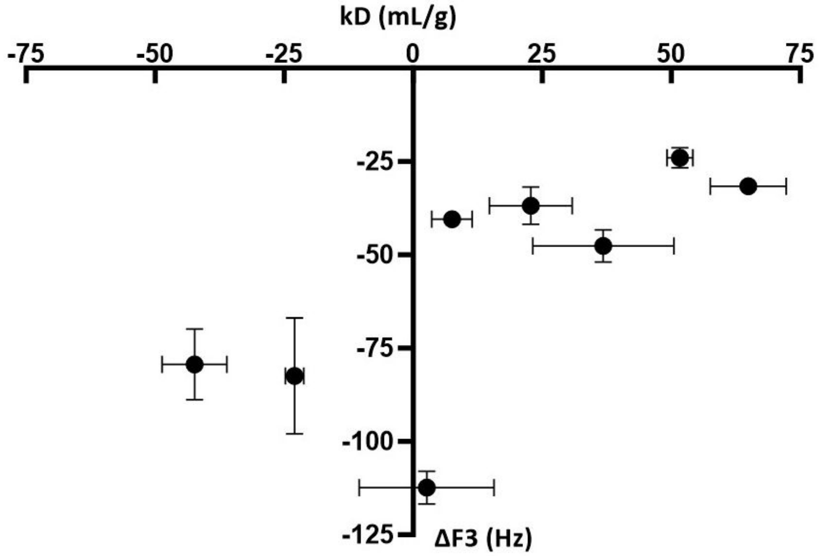

Supplementary Figure 9. Frequency changes associated with the loosely interacting layer, Δ*f* versus the Diffusion Interaction Parameter, kD-DLS for all eight antibodies studied

Supplementary Table 2. Summary of the statistical analysis of correlations between Δf3 and kD-DLS, as well as ΔD3 and kD-DLS, calculated for all eight mAbs studied [In all cases, there is a strong correlation, and this correlation is statistically significant in three out of the four comparisons (significance level of 0.05). Overall, Δ*f*3 shows a stronger correlation with kD-DLS compared to ΔD3 and is, therefore, our preferred metric for assessing self-association.]

	**Spearman correlation coefficient**	**P-value (Spearman)**	**Pearson correlation coefficient**	**P-value (Pearson)**
Δf_3 and kD-DLS	0.809	0.027	0.714	0.046
ΔD_3 and kD-DLS	-0.619	0.118	-0.735	0.038

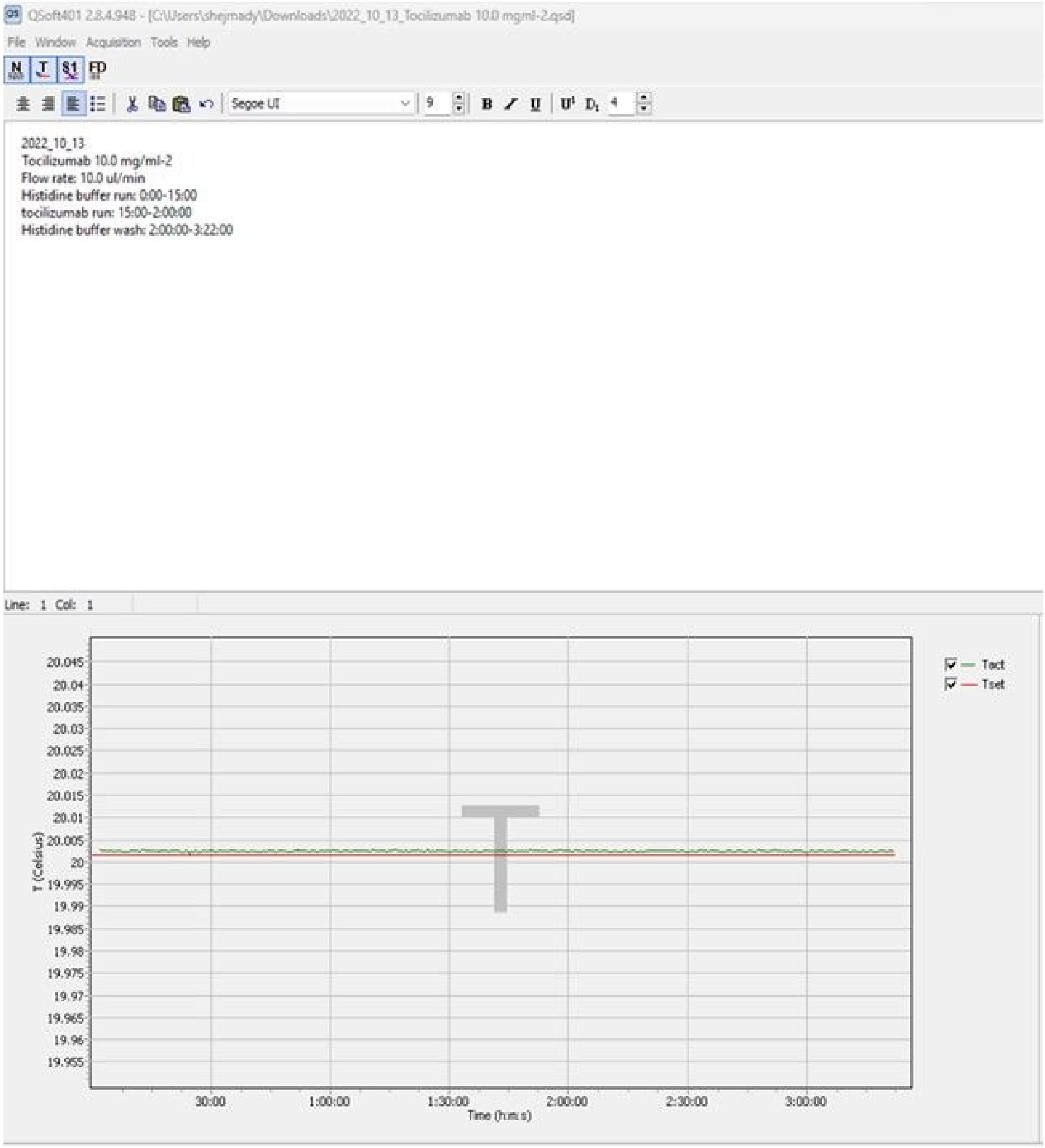

Supplementary Figure 10. Temperature variability during a representative QCM-D measurement (The red line indicates the set temperature (20°C), while the green line represents the actual temperature measured at the sample sensor interface.)
